# The protective mechanism of Dehydromiltirone in diabetic kidney disease is revealed through network pharmacology and experimental validation

**DOI:** 10.3389/fphar.2023.1201296

**Published:** 2023-08-23

**Authors:** Yanzhe Wang, Yuyuan Liu, Sijia Chen, Fengqin Li, Yue Wu, Xinmiao Xie, Nan Zhang, Chuchu Zeng, Linnan Bai, Mengshi Dai, Ling Zhang, Xiaoxia Wang

**Affiliations:** ^1^ Department of Nephrology, Tongren Hospital, School of Medicine, Shanghai Jiao Tong University, Shanghai, China; ^2^ Department of Nephrology, Suzhou Municipal Hospital, The Affiliated Suzhou Hospital of Nanjing Medical University, Suzhou, Jiangsu, China; ^3^ Department of Nephrology, Sir Run Run Shaw Hospital, Zhejiang University School of Medicine, Hangzhou, China; ^4^ Department of Geriatrics, Huashan Hospital, Fudan University, Shanghai, China; ^5^ Department of Obstetrics and Gynecology, Chengdu Women’s and Children’s Central Hospital, School of Medicine, University of Electronic Science and Technology of China, Chengdu, Sichuan, China

**Keywords:** diabetic kidney disease, network pharmacology, molecular docking, Dehydromiltirone, PIK3CA

## Abstract

**Background:** *Salvia miltiorrhiza* (SM) is an effective traditional Chinese medicine for treating DKD, but the exact mechanism is elusive. In this study, we aimed to investigate and confirm the method underlying the action of the active components of SM in the treatment of DKD.

**Methods:** Renal tissue transcriptomics and network pharmacology of DKD patients was performed to identify the active components of SM and the disease targets of DKD. Next, the point of convergence among these three groups was studied. Potential candidate genes were identified and analyzed using Gene Ontology (GO) and the Kyoto Encyclopedia of Genes and Genomes (KEGG). The component-target networks were modelled and visualized with Cytoscape. In addition, docking studies were performed to validate our potential target predictions. Lastly, *in vitro* and *in vivo* experiments were performed to understand the role of Dehydromiltirone (DHT), the active component of SM, in the phenotypic switching of mesangial cells.

**Results:** Transcriptomics of DKD patients’ renal tissues screened 4,864 differentially expressed genes. Eighty-nine active components of SM and 161 common targets were found. Functional enrichment analysis indicated that 161 genes were enriched in apoptosis, the PI3K-AKT signaling pathway, and the AGE-RAGE signaling pathway in diabetes complications. Molecular docking and molecular dynamic simulations show that DHT can bind to functional PIK3CA pockets, thereby becoming a possible inhibitor of PIK3CA. *In vitro* study demonstrated that DHT reduced the expression of phenotypic switching markers α-SMA, Col-I, and FN in HMCs by downregulating the over-activation of the PI3K-AKT signaling pathway through the inhibition of PIK3CA. Furthermore, the DKD mouse model confirmed that DHT could reduce proteinuria and improve glomerular hypertrophy *in vivo*.

**Conclusion:** DHT was identified as the key active component of SM, and its therapeutic effect on DKD was achieved by inhibiting the phenotypic switching of mesangial cells via the PIK3CA signaling pathway.

## 1 Introduction

Diabetic kidney disease (DKD) is a common persistent impediment of diabetes (Barrera-Chimal et al., 2022). It is also essential for end-stage renal disease (ESRD) (Koye et al., 2018). The pathogenesis of DKD is complex, involving metabolic disorders, hemodynamic changes, inflammatory response, cytokines, oxidative stress, genetic factors, autophagy, apoptosis and other factors (Fu et al., 2019; Tuttle et al., 2022). The currently available DKD therapies such as DPP-4 inhibitor (Gupta and Sen, 2019), SGLT-2 inhibitor (DeFronzo et al., 2021), ROCK inhibitors (Komers et al., 2011), ACEi and ARBs have been shown effective in combating DKD, but they could not completely prevent or reverse the progression of DKD (Kato and Natarajan, 2014; Wang et al., 2021). The main pathological change in the initial stage of DKD is the phenotypic switching of human mesangial cells (HMCs), which present with HMCs hypertrophy and extracellular matrix aggregation (Avraham et al., 2021). Its continuous progress will result in increased glomerular hypertrophy, thickened basement membrane, diffuse glomerulosclerosis, and eventually a progression to ESRD. Therefore, it is essential to develop drugs that delay the phenotypic switching of HMCs. Traditional Chinese Medicine (TCM) has recently attracted extensive attention in life sciences (Tang et al., 2021). TCM is a traditional Asian treatment method and a multi-level, multi-pathway, and multi-target (Huang et al., 2021) alternative medical practice to the Western medical system. The natural active molecules in TCM are a crucial source of new drug research and development (Yao et al., 2021).

*Salvia miltiorrhiza* (SM), a traditional Chinese herbal medicine, was first recorded in “Shen Nong’s Herbal Classic.” SM activates blood, removes stasis, cools blood, and eliminates carbuncles (Chinese Pharmacopoeia Commission, 2020). SM is a natural herb that pertains to the genus Sage in the family Lamiaceae. Studies have found more than 100 kinds of components in SM, including diterpene quinones, phenolic acids, danshen lactones, alkaloids, etc. The effective components of SM are generally divided into water-soluble and lipophilic parts (Shi et al., 2019). SM is an effective drug for improving the clinical symptoms of DKD (Nie and Li, 2018; Shen et al., 2020). Among the clinical prescriptions for treating DKD in China, SM is the second most frequently used medication (5.46%) (Lin et al., 2020). The possible mechanisms of SM in treating DKD include maintaining blood glucose balance, anti-oxidation stress response, regulating cytokines, inhibiting inflammatory response, improving renal perfusion, regulating blood lipid balance, improving metabolic memory and hypoxia status, etc (Xie et al., 2021). However, the specific mechanism by which SM improves phenotypic switching in HMCs has not been elucidated.

Network pharmacology is a new approach to drug design that combines pharmacology with systems biology. It is a new pharmacological research strategy with the idea of a “disease gene target drug” network regulation mechanism (Yildirim et al., 2007; Li et al., 2022a). SM is a complex pharmacological mixture having several components and multiple targets. Therefore, this study combined microarray and network pharmacology to screen possible SM targets for DKD and constructed a multi-objective network of SM to discover further the effective components and its key targets through bioinformatics. *In vitro* and *in vivo* experiments were performed to examine the specific mechanism of DHT on DKD. Our research aims to identify the effective active components and key targets of SM in treating DKD to provide a new strategy for preventing and treating DKD.

## 2 Materials and methods

### 2.1 Ethics statement

The ethics committee of Tongren Hospital, SJTUSM (Shanghai Jiao Tong University, School of Medicine) approved this research (Ethical Approval No. 2017-13). All research involving humans was performed with the patients’ consent. Animal procedures were conducted per the “Guide for the Care and Use of Laboratory Animals” (National Institutes of Health).

### 2.2 The collection and histopathological assessment of renal tissue

Patients were recruited from Tongren Hospital, SJTUSM. All study participants signed a written informed consent form. Renal cortexes were taken from patients with DKD diagnosed by clinical features and pathological changes in renal biopsies (Akhtar et al., 2020). Controls were healthy renal cortex samples taken from patients with renal carcinoma experiencing nephrectomy. Renal tissues, including periodic acid-Schiff (PAS), were obtained following renal biopsy or surgery for histopathological evaluation. Finally, based on the pathological results, the mRNA from 3 frozen DKD tissue samples and 3 control samples was used in the microarray assay.

### 2.3 Transcriptomic analysis

Total RNA from the renal cortex tissue was extracted following the TRIzole method (Invitrogen, Carlsbad, CA). Subsequently, the quantity and integrity of the RNA was measured using the NanoDrop 8,000 (Thermo Scientific) and the Agilent Bioanalyzer 2,100 (Agilent Technologies), respectively. Refer to the Affymetrix manual for cRNA related experiments (Campus IFOM IEO, Italy). Samples were hybridized onto a Human Clariom D gene chip (Affymetrix, Santa Clara, California). The R statistical software pre-processed imported raw CEL files, and the Robust Multichip Average algorithm under the oligo package was used to normalize the data. The raw data were investigated using the transcriptome analysis console software (Applied Biosystems, Foster City, USA). A significant (*p*-value < 0.05) absolute fold change in gene expression of ≥ 1.5 indicated differentially expressed genes (DEGs). The R software (heatmap V1.0.12 and ggplot2 V3.3.6) was used to generate heatmaps and volcano plots of significant DEGs (Tian et al., 2021).

### 2.4 The analysis of the pharmacology network

#### 2.4.1 Collection of the active SM components and their potential targets

Screening was conducted via the TCMSP database (https://tcmspw.com/tcmsp.php) to identify the active components of SM. To identify the absorptive, distributive, metabolic, and excretory (ADME) properties of the potentially active components of SM, oral bioavailability (OB), drug-likeness (DL), and drug half-life (HF) were determined. Components with an OB ≥ 20% and DL ≥ 0.18 were chosen for further study. PubChem (https://pubchem.ncbi.nlm.nih.gov/) was used to recognize the chemical structures of the screened active SM components. Target prediction using the BATMAN (http://bionet.ncpsb.org/batman-tcm/) and Swiss Target Prediction database (http://swisstargetprediction.ch/) was performed on components with available chemical structures (Daina et al., 2019). The species was restricted to “*Homo sapiens*,” and the UniProt ID of predicted targets was taken from the UniProt database.

#### 2.4.2 Collection of DKD-related genes

DKD-related genes were identified from many databases, including GeneCards (homepage https://www.genecards.org/), OMIM (homepage https://omim.org/), PharmGKB (homepage https://www.pharmgkb.org/), TTD (homepage http://db.idrblab.net/ttd/), DisGeNET (homepage https://www.disgenet.org), and DrugBank (homepage https://go.drugbank.com). Keywords used to search the databases included “diabetic kidney disease” and “diabetic nephropathy.” The findings were reviewed, de-duplicated, and classified as DKD-related targets. Finally, we visualized the above results using the Venn Diagrams package (Version 1.11).

#### 2.4.3 Common targets acquisition and functional enrichment analysis

The common DEG targets identified in the transcriptomic analysis, the potential targets of the active components, and the DKD-related genes were recognized and visualized by a Venn diagram. GO and KEGG enrichment analysis via the clusterProfiler R package was performed to examine the biological function of the common targets. The ggplot2 R package was used to visualize the results. A threshold of *p*-value < 0.05 identified key GO and KEGG pathways.

#### 2.4.4 Common targets PPI network building and identification of Hub genes

A protein-protein interaction (PPI) network (the cut-off standard as a combined score > 0.4) of the common targets was built using the STRING database (https://string-db.org) and visualized using the Cytoscape software (version 3.9.1). The Network Analyzer Tool Kit in Cytoscape was used to analyze the PPI network topology parameters (such as degree distribution, betweenness, and closeness) between the active components and targets. The nodes and edges in the network represent proteins and protein-protein interactions, separately. Although the edge identifies proteins that jointly contribute to a common function, it does not imply that the proteins are physically bound to each other. Therefore, a thicker edge corresponds to greater confidence. Molecular Complex Detection (MCODE), a plug-in in the Cytoscape software, was applied to identify significant modules, the threshold was set as 1. a-degree cut-off = 2, 2. node score cut-off = 0.2, 3. k-core = 4, 4. max. Depth = 100 as the criterion. The seed nodes identified by MCODE were considered the Hub genes.

#### 2.4.5 Network building of “drug-target-pathway”

Cytoscape software was used to visualize the key component-target-pathway enrichment network. The network topology parameters were determined using Network Analyzer. According to degree, betweenness, and closeness, the active components were identified and primed for molecular docking.

#### 2.4.6 Molecular docking and molecular dynamics simulation

The Protein Data Bank (PDB, http://www.rcsb. org/) was used to identify the structure of the active SM components and the 3D crystal structure of the possible target protein. The structure was modified, and molecular docking was performed using the AutoDockTools (Version 1.5.7) software. AutoDock Vina determined the binding energy and sites (Trott and Olson, 2010). The resulting predicted models were stored in PDB file format and visualized using the PyMOL 2.5.2 software. A binding affinity value < 0 kcal/mol suggests that the components bind to the targets effectively. To determine the dynamic interaction between DHT and the proteins (PIK3CA, 7JIU), molecular dynamics simulation (MDS) was performed by using AMBER 18 software. The force field used for the protein and the component was AMBERff14SB and GAFF2, respectively (Wang et al., 2004). TIP3P was selected as the solvent model, and the protein was placed in a cubic water box with a distance of 10 Å, between the edge of the box and the protein molecule. Sodium chloride (NaCl) was included to stabilize the charge of the system. The system’s energy was optimized using the 2500-step steepest descent and conjugate gradient method. The system’s temperature was slowly increased from 0 Kelvin (K) to 298.15 K by 200 ps heating. When the temperature got 298.15K, the density balance of 500 ps was achieved with regular boundary conditions. Finally, a 50 ns MDS was performed.

#### 2.4.7 Free energy binding and component computation

MDS trajectories between 45 and 50 ns were computed by the MM/GBSA method to evaluate the binding free energy between protein and component (Genheden and Ryde, 2015). The exact formula is as follows:

$${\Delta G}_{bind}={\Delta G}_{complex}\;–\;\left({\Delta G}_{receptor}+{\Delta G}_{ligand}\right)={\Delta E}_{internal}+{\Delta E}_{VDW}+{\Delta E}_{elec}{+\Delta G}_{GB}+{\Delta G}_{SA}$$

In this study, ${\Delta E}_{internal}$ represented internal energy, ${\Delta E}_{VDW}$ represented van der Waals action, and ${\Delta E}_{elec}$ represented electrostatic interactions. The internal energy included bond energy (Ebond), angle energy (Eangle), and torsion energy (Etorsion); ${\Delta G}_{GB}$ and ${\Delta G}_{GA}$ were jointly described as solvation free energy. Among them, GGB was the polar solvation free energy and GSA was the non-polar solvation free energy (Nguyen et al., 2013).

### 2.5 Experimental validation

#### 2.5.1 Cell and reagents

A human mesangial cell line (HMC cell line, FH0241) was obtained from FuHeng Biology Co. (Shanghai, China). DHT (116064-77-8, purity ≥ 98%) was acquired from Krre Technology Co. (Beijing, China). Mannitol (69-65-8) was obtained from Macklin Biochemical Co. (Shanghai, China). The following antibodies: GAPDH (1:1000, ab8245), α-smooth muscle actin (1:1000, ab7817), Collagen I (1:1000, ab260043), Fibronectin (1:1000, ab2413), PI3KCA (1:1000, ab40776), PI3K (1:1000, ab191606), p-PI3K (1:1000, ab182651), AKT (1:1000, ab179463), and p-AKT (1:1000, ab192623) were bought from Abcam Co. (Cambridge, United Kingdom). Goat Anti-Rabbit IgG H&L (HRP, A0208), Rabbit Anti-Mouse IgG H&L (HRP, A0216), and Rabbit Anti-Mouse IgG H&L (Alexa Fluor 488, A0428) were purchased from Beyotime (Shanghai, China). DAPI Staining Solution (C1006) and Cell Counting Kit-8 (C0038) were also obtained from Beyotime (Shanghai, China). Glucose, Trypsin, Dulbecco’s Modified Eagle’s Medium (DMEM), and fetal bovine serum (FBS) were purchased from GIBCO. The Mouse Albumin ELISA Kit (ab207620) was purchased from Abcam.

#### 2.5.2 Cell culture, treatment, and transfection

HMCs were grown in DMEM (Gibco, USA) enhanced with 10% FBS (Gibco, USA), 100 U/mL penicillin (Amresco, USA), and 100 U/mL streptomycin (Amresco, USA), and incubated with 5% CO_2_ at 37°C. The growth state of HMCs was observed under a microscope. Cell passage was carried out when the growth density reached 90%, and the 3rd to 6th-generation cells were used for the experiment. After 24 h of cell passage, HMCs were starved to a serum-free medium for 24 h to synchronize. Next, the cells were grown in a medium containing 5.5 mM D-glucose (NG), 5.5 mM D-glucose and 24.5 mM mannitol (MA) or 30 mM D-glucose (HG) for 48 h to determine the effect high glucose has on them. To determine the role of PIK3CA in phenotype remodeling of HMCs due to high glucose, the nonspecific control (NC) and siRNA duplexes (GCG​AAA​TTC​TCA​CAC​TAT​T) targeting human PIK3CA mRNA (GenBank accession no. NM_006218) were designed and produced by Guangzhou RiboBio Company (Guangzhou, China). HMCs were plated in an antibiotic-free growth medium at 30%-40% confluency, roughly 24 h before transfection. Lipofectamine RNAiMAX (Invitrogen, USA) transfected the RNA oligonucleotides at a concentration of 50 nM, following the manufacturer’s procedure. Additional treatment ensued 48 h after transfection.

#### 2.5.3 Cell viability assay

A CCK-8 assay was used to analyze the inhibitory effect of DHT on the viability of HMCs. HMCs (5× 10^3^ cells/well) were inoculated into 96-well plates. After incubation, the cell culture medium was substituted with fresh medium containing various concentrations (0.1, 1, 5, 10, 100 µM) of DHT and incubated for 48 h. Next, 10 μL of CCK-8 solution was added to every well, after which the cells were incubated at 37°C for 1 h. Each well’s optical density (OD) was calculated at 450 nm using a spectrophotometer 1510 (Thermo Fisher Scientific, USA).

#### 2.5.4 Immunofluorescence staining

HMCs were plated on μ-Slides (#80297; Ibidi, Martinsried, Germany) and treated with 4% paraformaldehyde for 30 min. After treatment, the cells were cleaned with PBS, permeabilized with 0.1% Triton X-100 for 10 min, and blocked with 10% donkey serum (G1217, Servicebio, China) at 23°C ± 2°C for 60 min. Subsequently, the cells and primary antibodies (1:500) were incubated overnight at 4°C. Next, the HMCs were cleaned using Tris-Buffered Saline Tween 20 (TBST) and incubated with the Alexa Fluor (1:500) for 1 h at 23°C ± 2°C. Finally, the nuclei were stained with DAPI (1:1000) for 5 min at room temperature. Immunofluorescence images were acquired with a Nikon ECLIPSE Ts2-FL (Nikon Instruments, Melville, NY).

#### 2.5.5 Western blot

RIPA lysate (Beyotime, Shanghai, China) was used to extract total proteins from the diversely treated HMCs and renal tissues of mice for western blot analysis. A bicinchoninic acid (BCA) protein assay kit (G2026, Servicebio, China) was used to determine protein concentration. Equivalent quantities of the protein samples were separated by 4%-12% SurePAGE (M00652, GenScript, USA), transferred to a PVDF membrane, and blocked with 5% BSA or dry milk in TBST for 1 h at 23°C ± 2°C. The primary antibodies (1:1000 dilution) were incubated 12-16 h at 4°C, the membranes were washed 3 times with TBST, and incubated for 1 h at 23°C ± 2°C with horseradish peroxidase-conjugated secondary antibodies (1:1000 dilution). The membranes were then incubated with enhanced chemiluminescence (ECL) reagent and imaged using the Tanon 5,200 luminescent imaging workstation. The ImageJ software was used to analyze the images. A strip solution washed membranes which needed to be imaged again. The representative experimental results were repeated three times.

#### 2.5.6 Animal grouping and intervention

Six-week-old male BKS-db/db and littermate db/m mice were obtained from Cavens Laboratory Animal Co., Ltd. (Changzhou, China, License No: SCXK 2016-0010) and raised in the SPF level barrier system of the Animal Laboratory of Hongqiao International Institute of Medicine, Tongren Hospital, SJTUSM. All experimental animals were fed in separate cages, with no more than three mice per cage. They ate and drank freely. Daily light exposure was for 12 h, the room temperature was 22°C ± 2°C, and the humidity was 50%-55%. At 8 weeks of age, the mice were randomly assigned into four groups, with 6 mice in each group, as follow: ①Con group (db/m mice treated with NS); ②DKD group (db/db mice treated with NS); ③DKD+DHT_L group (db/db mice treated with 10 mg/kg DHT); ④DKD+DHT_H group (db/db mice treated with 20 mg/kg DHT). DHT was injected intraperitoneally every 3 days for 16 weeks. Every 2 weeks the fasting blood glucose level (FBG) in all animals was assessed using an Accu-Chek Advantage Meter (Roche, Mannheim, Germany). Non-fasting mice were placed in metabolic cages at 12 and 24 weeks of age, respectively, and urine samples were collected for 24 h. After fasting for 12 h, 1% pentobarbital was used to euthanize the mice at 24 weeks. Blood samples were obtained from the horn vein, and after centrifugation at 4°C 3500 g for 10 min, serum was obtained.

#### 2.5.7 Biochemical assessment and histopathological analysis

Urinary albumin concentration was measured using the mouse albumin ELISA kit (ab207620, Abcam). This value was multiplied by each mouse’s 24 h urine output to determine the 24 h urinary albumin excretion. The levels of serum creatinine (SCr) and blood urea nitrogen (BUN) in serum were determined by Servicebio Technology Co. (Wuhan, China). The mouse kidneys were fixed overnight in 10% formalin at 23°C ± 2°C and surrounded with paraffin. Next, 2–3 μm sections were stained with hematoxylin-eosin (H&E), periodic acid-Schiff (PAS), Masson and periodic acid-silver methenamine (PASM). The quantification of glomerular areas was performed according to an earlier reported method (Linnan et al., 2021). For IHC staining, the tissue sections of α-SMA (Affinity, AF1032, 1:400) were performed as earlier described (Liu et al., 2022).

#### 2.5.8 Statistical analyses

GraphPad Prism 7 (Version 7.00) was used to analyze and plot data. Data was gathered by Adobe Illustrator CS6 (Version 16.0.0) and the results were provided as means ± SEM. For all data comparisons, one-way ANOVA and two-tailed unpaired Student’s t-test were used to perform the statistical analysis. Statistical significance was indicated by a *p*-value < 0.05.

## 3 Results

### 3.1 Acquisition of common targets between SM and DKD

#### 3.1.1 Differentially expressed genes identified by transcriptome analysis

**FIGURE 1:**
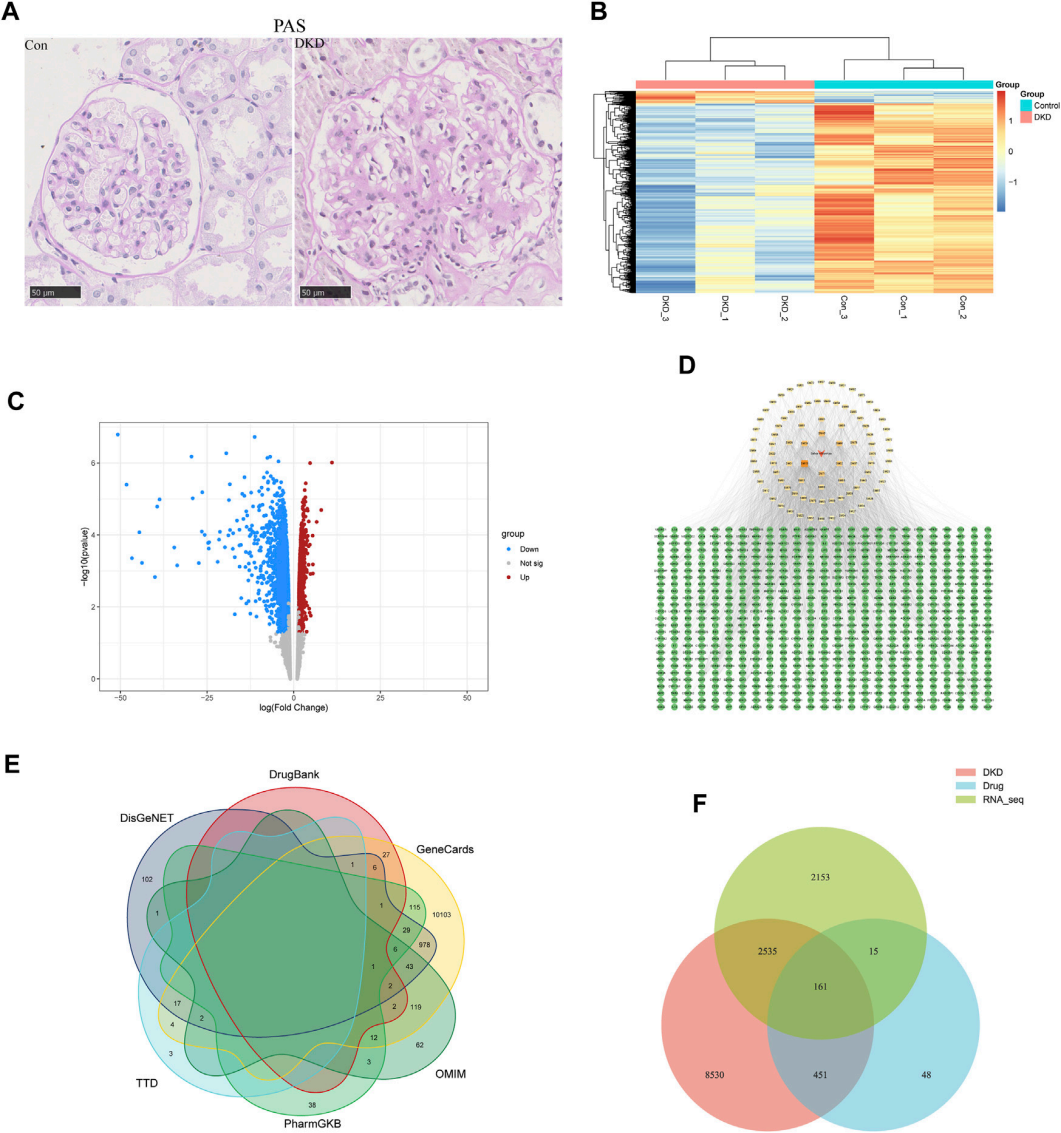
Acquisition of common targets between SM and DKD. **(A)** PAS coloring of controls and patients with DKD in kidney (n = 5), Scale bar: 50 μm, original magnification ×400. **(B)** Heatmap analysis of differentially expressed genes (DEGs) in transcriptome of clinical samples. Upregulated DEGs were shown in orange, while downregulated DEGs were shown in blue. **(C)** transcriptome volcano plot analysis of clinical samples. Red dots represented upregulated genes and blue dots represented downregulated genes. Gray dots represented genes with no significant differences in expression. **(D)** Active component targets of SM and it is network analysis. A total of 89 active components and 675 predicted targets were selected. The red triangle represented SM, the yellow square represented the active components, and the green circle represented the predicted targets. The color of the active components gradually darkens as the Degree increases. **(E)** Network pharmacology of DKD-related genes. **(F)** Venn diagram of targets on the above three data sets.

To identify differentially expressed genes (DEGs) in DKD, renal tissues from type 2 DKD patients with Mogensen stage 3 and renal biopsy results that met the diagnostic criteria of DKD, were selected as the research objects. In addition, healthy renal tissues from non-diabetic patients with renal tumor resection were selected for the control group (Tervaert et al., 2010). In the control group, glomeruli staining using PAS revealed opened capillary loops without increased matrix. In contrast, PAS staining of glomeruli in the DKD group showed a widening of the mesangial area and increased matrix (400X) (Figure 1A). According to the pathological results, DKD tissues from three cases and three controls were selected for transcriptome analysis. From these, 4,864 differentially expressed mRNAs were identified (FC ≥ 1.5, *p* < 0.05), of which 297 were upregulated, and 4,567 were downregulated (Figures 1B, C).

#### 3.1.2 Active components and SM targets

ADME screening identified 89 active SM components (Supplementary Table S1). Genes were regarded as potential drug-targeted genes if the BATMAN Score cut-off was ≥ 10 and the SwissTargetPrediction Probability score was > 0. Overall, 675 genes were screened as drug-targeted genes (Supplementary Table S2). Cytoscape 3.9.1 was used to construct the active components–target network (Figure 1D).

#### 3.1.3 Acquisition of the disease targets of DKD and the common target

Using “diabetic kidney disease” or “diabetic nephropathy” as keywords, the GeneCards, OMIM, PharmGKB, TTD, DisGeNET, and DrugBank databases identified 11468, 253, 205, 27, 1189, and 95 disease targets, respectively. After removing duplicates, 11677 disease targets were identified (Figure 1E). In addition, Venn analysis was performed on the above three data sets, and 161 common targets were gained after intersection (Figure 1F).

#### 3.1.4 Functional enrichment analysis of common targets

**FIGURE 2:**
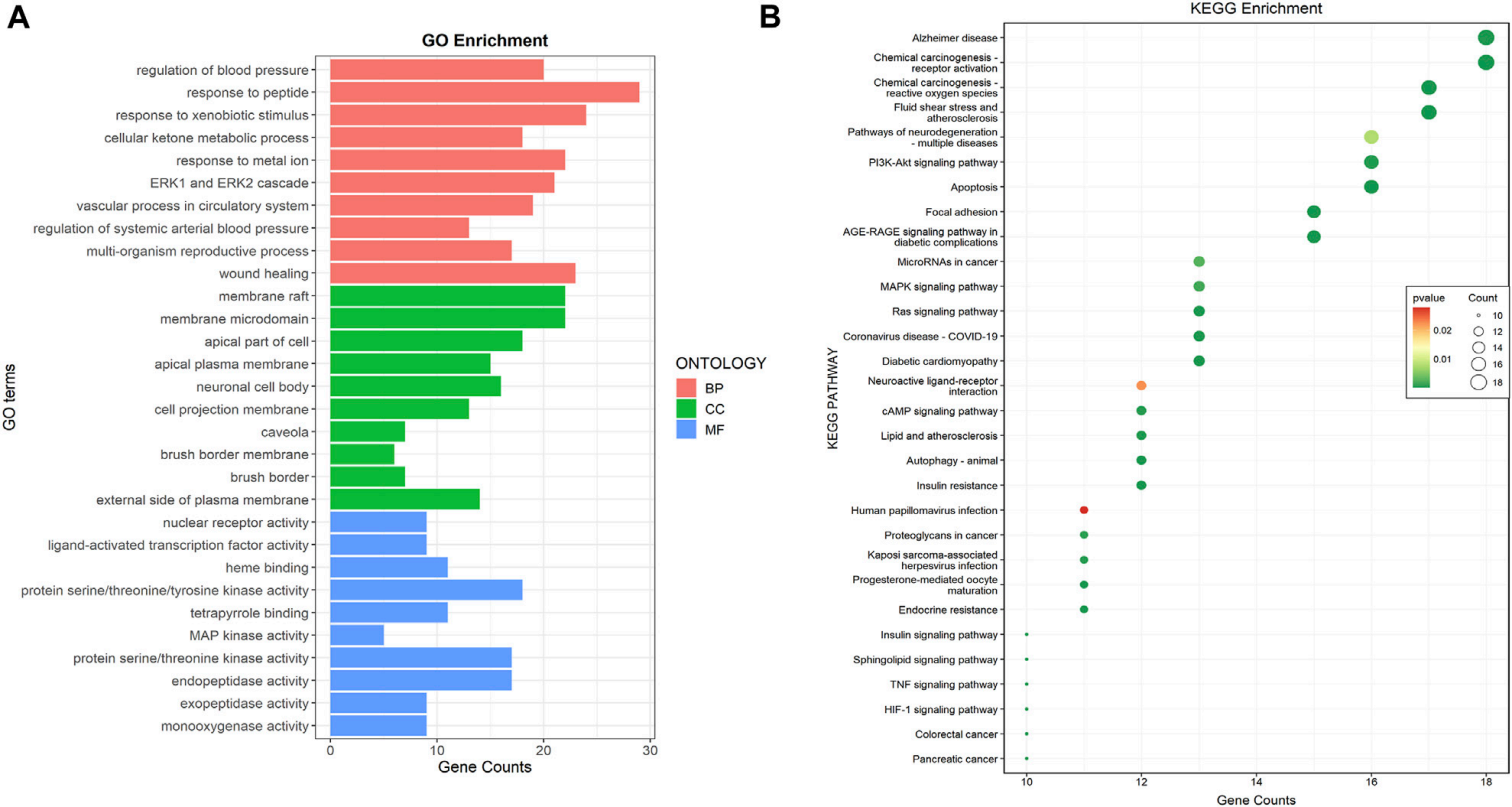
Common targets functional enrichment analysis. **(A)** GO analysis on common targets (top 10). Red represented biological process, green represented cellular component, and green represented molecular function. **(B)** KEGG pathway exploration of common targets (top 30). The size of the bubble indicated the pathway count, while colors indicated the significance of *p*-value.

The GO enrichment analysis revealed 1568 GO terms, of which 1405 were associated with biological processes, including “regulation of blood pressure,” “response to peptide,” and “response to xenobiotic stimulus.” Forty-four GO terms were related to cellular components, including “membrane raft,” “membrane microdomain,” and “apical part of a cell.” One hundred and nineteen GO terms were related to molecular functions, including “nuclear receptor activity,” “the ligand-activated transcription factor activity,” and “protein serine/threonine/tyrosine kinase activity” (Figure 2A). KEGG enrichment analysis revealed 134 signaling pathways, among which apoptosis, the PI3K-AKT signaling pathway, and focal adhesion were the most enriched pathways related to DKD (Figure 2B).

### 3.2 Establishment of Hub genes and key component of SM

#### 3.2.1 PPI network formation of common targets and the discovery of Hub genes

**FIGURE 3:**
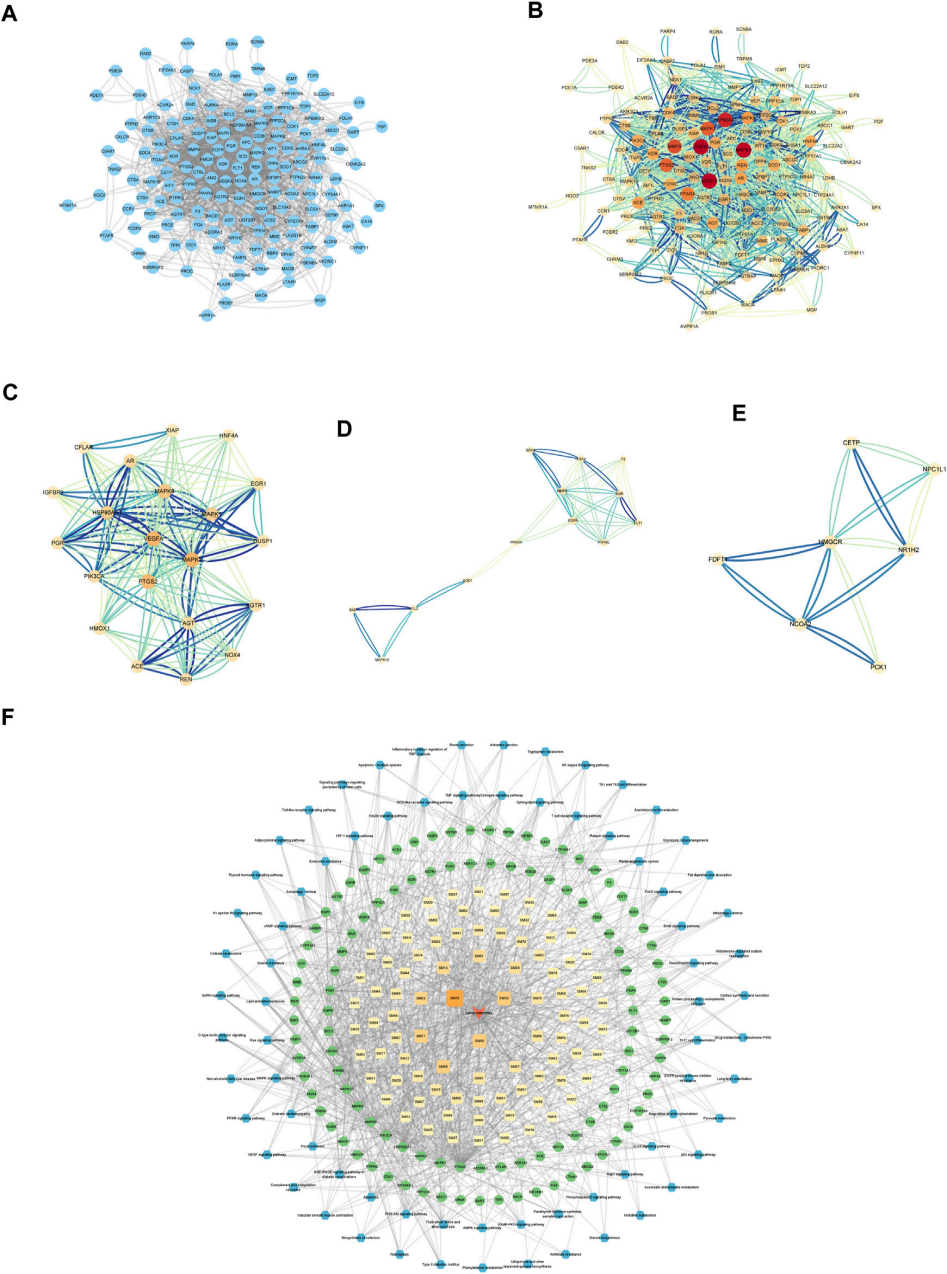
Establishment of Hub genes and key component of SM. **(A)** PPI web of common targets. **(B)** PPI network analysis. The orange circle represented the common targets, and its size represented the Degree value. **(C)** Cluster1 analysis by MCODE (Score:10.8, Seed: PIK3CA). **(D)** Cluster2 analysis by MCODE (Score:4.667, Seed: PPARA). **(E)** Cluster3 analysis by MCODE (Score:4, Seed: CETP). **(F)** Visual analysis of ingredient–common target-pathway network. Yellow squares symbolize the calculated components, while green circulars symbolize the targets, and blue octagons symbolize the calculated pathways. The color and magnitude of the border are settled conforming to the degree value.

PPI networks were created and analyzed as described in the Methods section. The network included 157 nodes and 1634 edges (Figures 3A, B). The MCODE Cluster analysis method analyzed the PPI network nodes to identify central target genes that play a vital role in the progression of DKD. As a result, the top three clusters were obtained: Cluster 1 (Score 10.8, Seed PIK3CA), Cluster 2 (Score 4.667, Seed PPARA), Cluster 3 (Score 4, Seed CETP) (Figures 3C–E).

#### 3.2.2 Network construction of “component–common target-pathway”

Visualizing selected KEGG signaling pathways related to the incidence and development of DKD, 89 active components that act on 108 targets and play a role in 74 signaling pathways were detected. This indicates that SM may delay the incidence and development of DKD through multi-components, multi-targets, and multi-pathways.

#### 3.2.3 Molecular docking and molecular dynamics simulation

**FIGURE 4:**
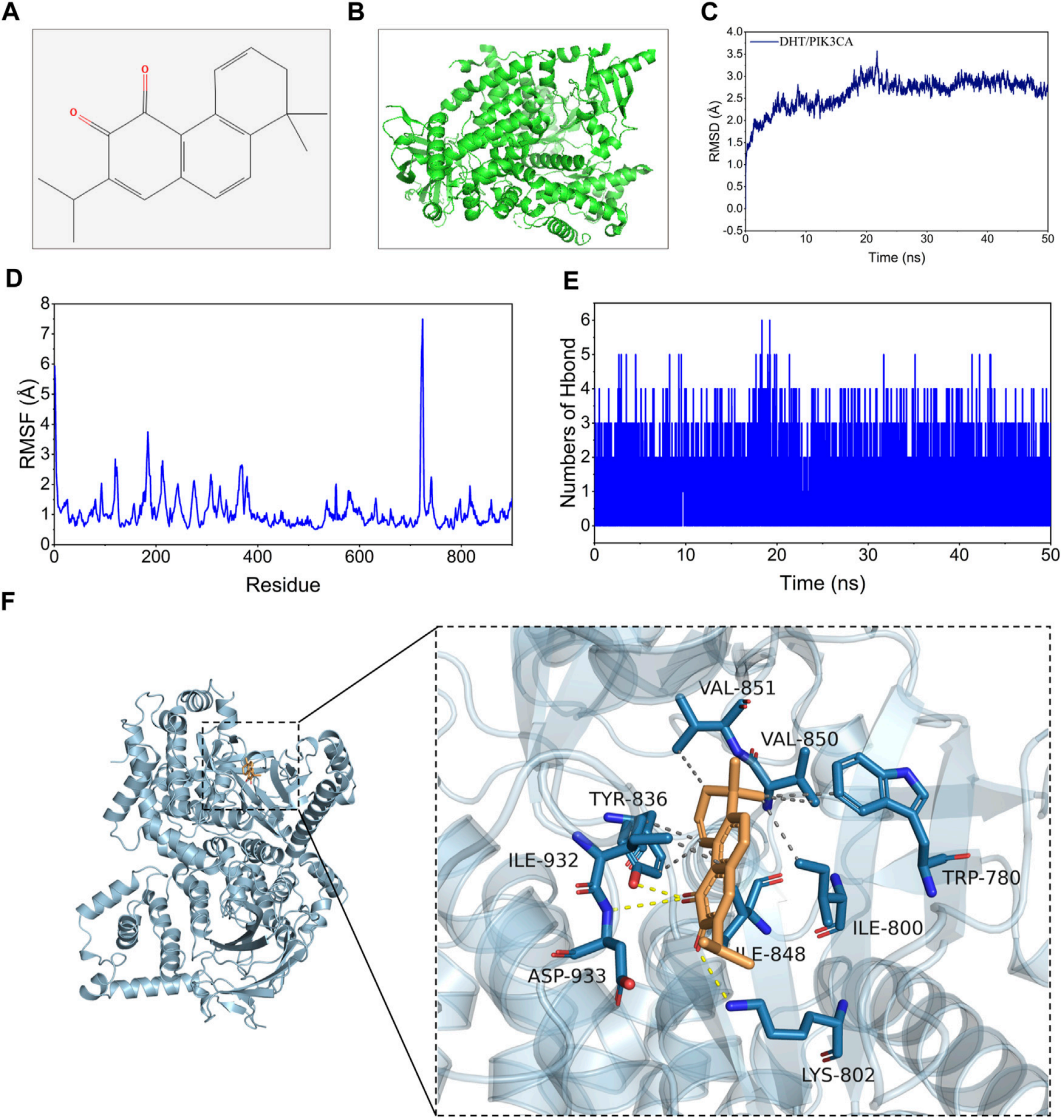
Molecular docking and MDS of DHT and PIK3CA. **(A)** Molecular formula of DHT. **(B)** Global graph of PIK3CA (7JIU). **(C)** RMSD profile of DHT with PIK3CA during through MDS. **(D)** The profiles of complex residues RMSF during 50 ns MDS. **(E)** Variations in hydrogen bond number between DHT and PIK3CA during MDS. **(F)** Binding mode of DHT and PIK3CA through MDS (left: global graph, right: local graph; the wheat color represents DHT, the yellow dotted line indicates hydrogen bonding, and the gray dotted line specifies hydrophobic interaction).

In this study, binding energy ≤ −5 kJ/mol was used as the screening criteria, and the top three active components by degree (DHT, Sclareol, and Aethiopinone) were chosen for molecular docking. The binding energy of DHT and PIK3CA was the lowest at −8.6 kJ/mol. Their molecular structure is shown in Figures 4A, B. According to the initial structure of the DHT-PIK3CA docking complex, MDS for 50 ns was carried out (Figure 4C). The RMSD of the DHT-PIK3CA complex attained a convergent state after 20 ns and fluctuated steadily around 3.5 Å. Additionally, the RMSF results of MDS showed that the RMSF of most residues was less than 2 Å, suggesting that the complex had minimal flexibility and good binding (Figure 4D). We examined the change in hydrogen bond number formed by DHT and PIK3CA proteins during molecular dynamics simulation (Figure 4E). During the simulation, between zero and four hydrogen bonds formed by DHT and PIK3CA. This indicates that DHT and PIK3CA had a certain number of stable hydrogen bonds. The molecular docking results reveal hydrogen bonding between DHT and LYS-802, ASP-933, and TYR-836 of PIK3CA, and hydrophobic interactions with ILE932, TYR-836, VAL-851, etc. (Figure 4F). The results suggest that DHT has a strong binding affinity with PIK3CA.

#### 3.2.4 Free energy binding and component computation

The binding energy of DHT and PIK3CA was calculated following the MM-GBSA method which accurately indicates the binding approach of small molecules and target proteins. Table 1 shows that the binding energy of DHT-PIK3CA was −28.64 ± 0.89 kcal/mol. Based on residue decomposition, van der Waals interaction is the primary contributor to DHT-PIK3CA binding, followed by non-polar solvation-free energy. This indicates that hydrophobic interactions may be the most important forces.

**TABLE 1:** Binding free energy and energy components predicted by MM/GBSA (kcal/mol).

System name	PIK3CA_DHT
Δ*E*_vdw_	−36.71 ± 1.08
Δ*E*_elec_	0.37 ± 2.83
ΔG_GB_	12.49 ± 3.89
ΔG_SA_	−4.79 ± 0.20
ΔG_bind_	−28.64 ± 0.89

ΔE_vdW_, van der Waals energy; ΔE_elec_, electrostatic energy; ΔG_GB_, electrostatic contribution to solvation; ΔG_SA_, non-polar contribution to solvation; ΔG_bind_, binding free energy.

#### 3.2.5 The effect of DHT on inhibiting phenotypic switching of HMCs through PIK3CA *in vitro*

**FIGURE 5:**
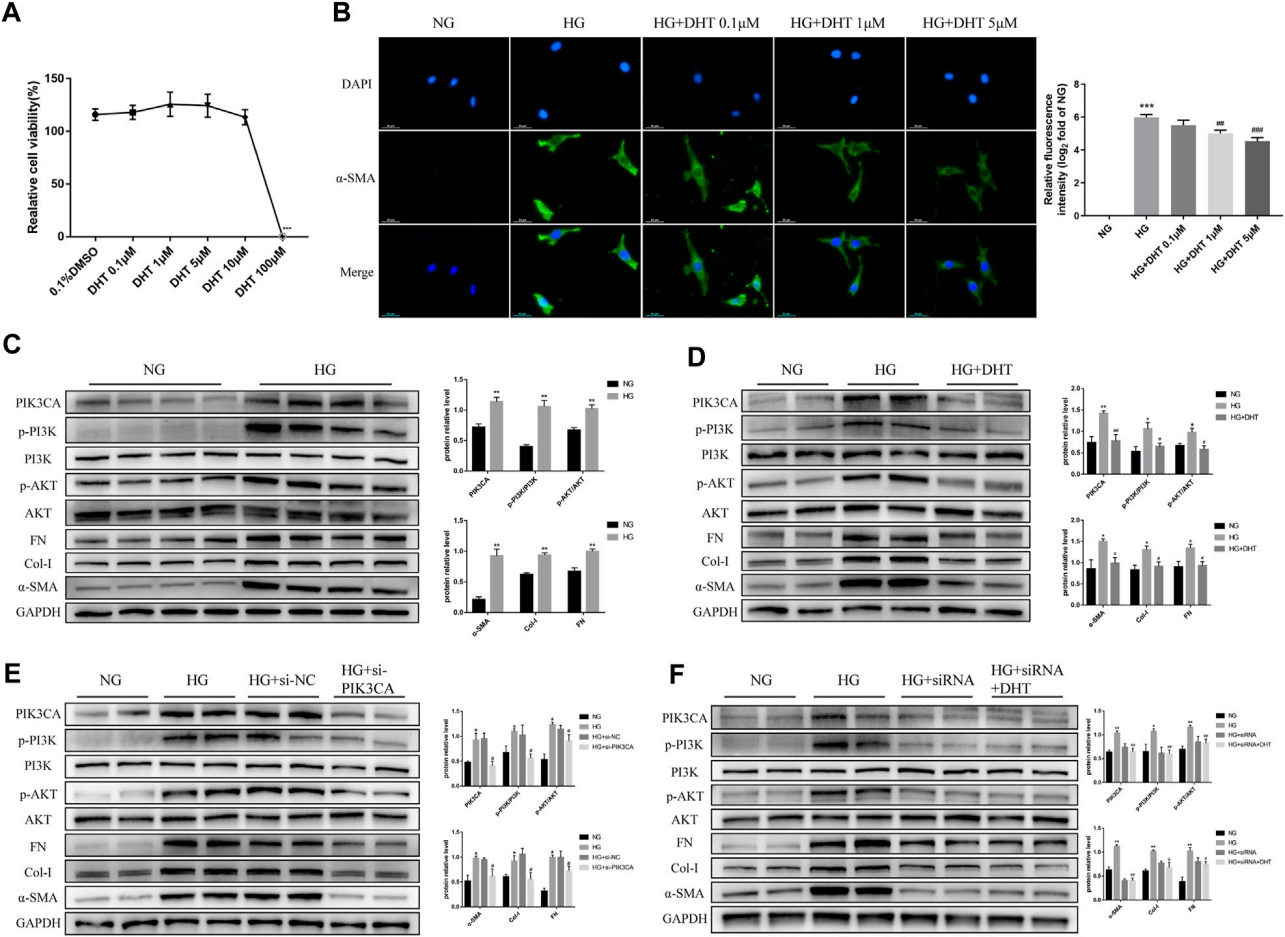
DHT inhibition of the phenotypic switching of HMCs through PIK3CA *in vitro*. **(A)** HMCs cultured in normal glucose treated with different DHT concentrations were detected by the CCK-8 method. ***p < 0.001, *versus* 0.1% DMSO (n = 5). **(B)** Immunofluorescence of HMCs induced by 30 mM glucose for 48 h and treated with 0.1 μM, 1 μM and 5 μM DHT and its semiquantitative analysis. Illustrative pictures of immunofluorescence staining for α-SMA (green) and DAPI (blue) (Scale bars: 50 μm) (n = 3). **(C–F)** Expression levels of the proteins PIK3CA, p-PI3K/PI3K, p-AKT/AKT, α-SMA, Col-I, and FN among HMCs induced by 30 mM glucose for 48 h and different intervention groups, followed by their semiquantitative analysis (n ≥ 3). *p < 0.05, *versus* Con; **p < 0.01, *versus* Con; ^#^ *p* < 0.05, *versus* HG; ^##^ *p* < 0.01, *versus* HG.

Next, we examined the mechanism of DHT in inhibiting phenotypic switching of HMCs *in vitro*. First, the safe dose of DHT in HMCs *in vitro* was determined using CCK-8. Results showed that a DHT concentration above 5 μM affected cell viability (Figure 5A) and significantly reduced the α-SMA green fluorescence signal induced by high glucose. In the subsequent study, 5 μM DHT was chosen as the intervention concentration (Figure 5B). There was no significant difference in the expression of phenotype switching marker proteins α-SMA, Col-I and FN between the MA group and the NG group (Supplementary Image S2). Further studies showed the expression of PIK3CA, p-PI3K/PI3K, p-AKT/AKT, and phenotypic switching marker proteins α-SMA, Col-I, and FN of HMCs were significantly increased after 48 h of high glucose induction (Figure 5C). After 5 μM DHT intervention, the expression of PIK3CA, p-PI3K/PI3K, p-AKT/AKT was obviously reduced, and the expression of α-SMA, Col-I, and FN was inhibited (Figure 5D). Inhibition of PIK3CA expression by siRNA inhibited the expression of PIK3CA, p-PI3K/PI3K, p-AKT/AKT, α-SMA, Col-I, and FN (Figure 5E). Based on siRNA-PIK3CA administration, the expression of the above-mentioned proteins was not significantly altered by DHT intervention (Figure 5F). These results suggest that DHT inhibits phenotypic switching of HMCs through PIK3CA regulation of the PI3K-AKT signaling pathway.

#### 3.2.6 DHT inhibited phenotypic switching of HMCs *in vivo*

**FIGURE 6:**
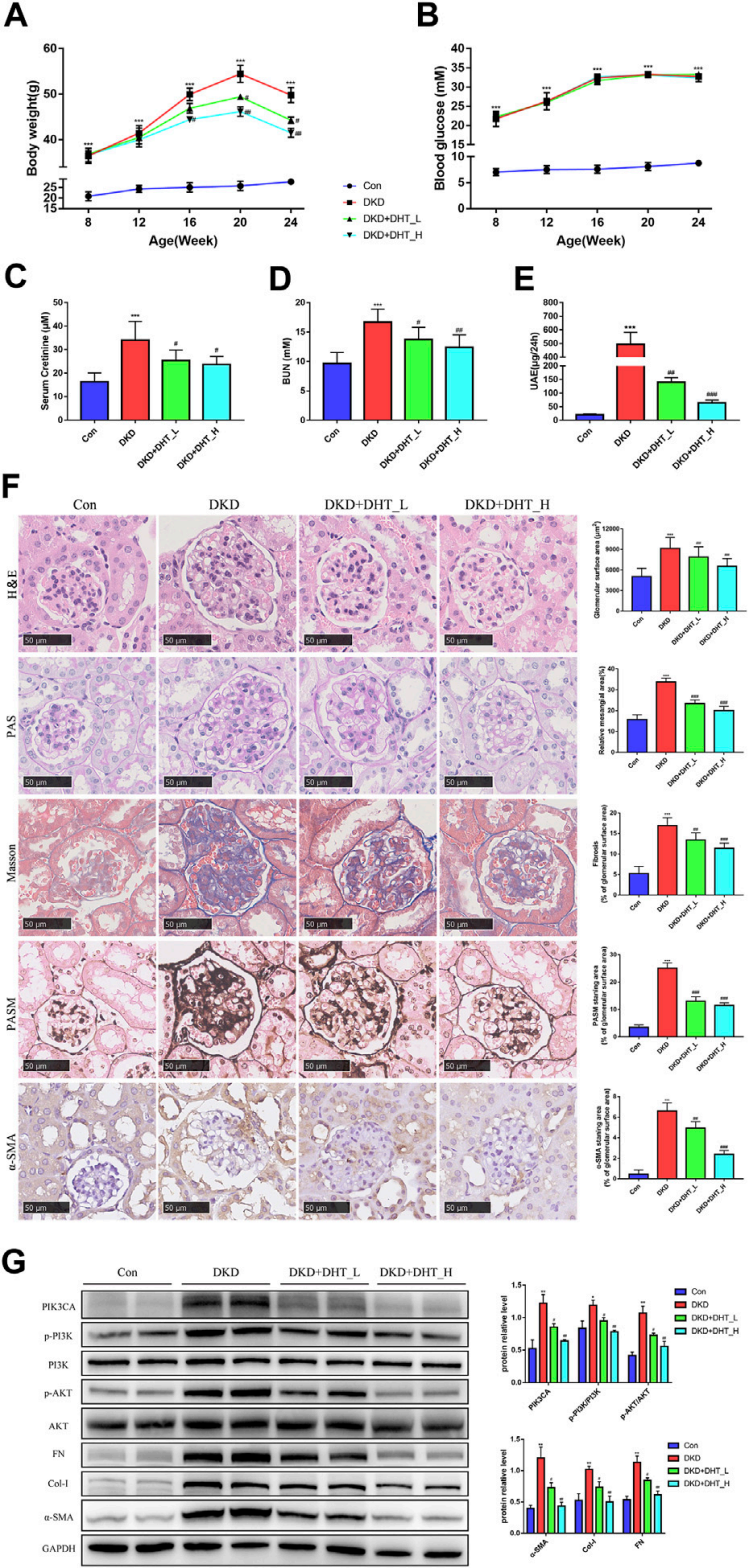
DHT inhibited phenotypic switching of HMCs *in vivo*. **(A)** Body weight of the animal model during the experimental period. **(B)** FBG of the animal model during the experimental period. **(C–E)** SCr, BUN and UAE levels in 24-week-old animal models. **(F)** Representative images of kidney tissues with H&E, PAS, Masson, PASM staining and a-SMA immunohistochemistry after DHT treatment for 16 weeks. Quantification of glomerular surface area, relative mesangial area, Masson, PASM and a-SMA staning area was shown as mean ± SEM (n = 6). Scale bar: 50 μm, original amplification X400. **(G)** Expression levels of the proteins PIK3CA, p-PI3K/PI3K, p-AKT/AKT, α-SMA, Col-I, and FN in DKD model mice (n = 3). *p < 0.05, *versus* Con; **p < 0.01, *versus* Con; ***p < 0.001, *versus* Con; ^#^ *p* < 0.05, *versus* DKD; ^##^ *p* < 0.01, *versus* DKD; ^###^ *p* < 0.001, *versus* DKD.

In addition, we performed an *in vivo* study using DHT. In the DKD mouse model, different concentrations of DHT could reduce body weight, SCr, BUN, and proteinuria, with high-dose DHT having a more significant effect (Figures 6A, C–E). Notably, DHT improved renal function in DKD mouse without causing changes in FBG (Figure 6B). H&E staining showed that glomeruli were significantly hypertrophy in the DKD group compared with the Con group, and the glomerular surface area was significantly reduced in the DHT treatment group (Figure 6F, H&E). PAS staining showed that the DKD+DHT_H group had significantly alleviated mesangial expansion and matrix proliferation compared with the DKD group (Figure 6F, PAS). Masson staining showed that glomerular fibrosis was significantly improved in the DHT treatment group compared with the DKD group (Figure 6F, Masson). PASM staining indicated that compared with the DKD group, the DHT treatment group could significantly alleviate the increase of collagen deposition in the mesangial area, and the high-dose DHT had a more significant effect (Figure 6F, PASM). The results of IHC indicated that the expression of a-SMA in the DHT treatment group was obviously lower than that in the DKD group (Figure 6F, α-SMA). Western Blot results indicated that DHT could inhibit the expression of PIK3CA, p-PI3K/PI3K, and p-AKT/AKT, and the expression of phenotypic switching marker proteins α-SMA, Col-I, and FN *in vivo* (Figure 6G).

## 4 Discussions

Unlike other diabetic microvascular and macrovascular downwards that happened to decline in the last 10–20 years, DKD occurrence is still constant (Gregg et al., 2014). Current clinical trial drugs for DKD, such as Aliskiren, have failed after the reports of non-fatal stroke, renal complications, hyperkalemia, and a higher-than-expected incidence of hypotension, among other adverse events (Lytvyn et al., 2020). Therefore, new therapies for DKD are urgently needed. In recent years, TCM research for DKD treatment has been on the ascendant. SM, also known as Danshen, is an ancestral, frequently-used Chinese remedy with a history of nearly 2000 years. The compound Danshen Dropping Pill was the first compound medicinal preparation from China to be accepted by the US Food and Drug Administration to undergo clinical trials (http://clinicaltrials.gov/:NCT01659580). It has already finished phase-III clinical trials in the US (Liao et al., 2019), which supports the safety of SM use. The effect of SM on DKD is noteworthy, as described in several publications. It has been reported that SM can reduce the risk of death due to DKD before dialysis (Guo et al., 2021). Animal studies have indicated that SM works well in STZ-induced diabetic rat kidney injury and regulates abnormal glucose and lipid metabolism (Xiang et al., 2019). Moreover, *in vitro* experiments revealed that Tanshinone IIA, one of the components of SM, inhibits hyperglycemia-driven apoptosis and inflammation of renal tubule epithelial cells by the modulation of transforming growth factor β1 (TGF-β1) (Li et al., 2022b).

Early pathological changes due to DKD include hypertrophy of mesangial cells, increased mesangial matrix, and enlarged glomerular volume (Alicic et al., 2017). HMCs are the main cells to be injured by DKD, and phenotypic switching of HMCs is the initial stage of DKD (Mason and Wahab, 2003). The induced cascade effect occurs as follows: HMCs express α-SMA, which can gain a myofibroblast character and significantly enhance the ability to synthesize extracellular matrix (ECM). Excessive ECM deposition leads to the above-mentioned pathological changes and is involved in the formation of early glomerulosclerosis, which eventually pushes towards renal function damage (Steffes et al., 1989). Still, little is known about how SM influences the HMCs phenotypic switching. In this context, the present study combined clinical sample transcriptomics and network pharmacology to determine the active constituents and key targets of SM, and then employed bioinformatics and mechanism studies to further elucidate how SM improves HMCs phenotypic switching.

Network pharmacology is a valuable implement to understand the complex relationships among diseases, natural products, and ingredients. In this paper, we recognized the Seed genes of top3 clusters from the PPI network as Hub genes through MCODE, namely PIK3CA, PPARA and CETP. The three active ingredients of SM (DHT, Sclareo, and Aethiopinone) had their molecular docking built to the Hub genes to test their chemical forces. The results, including molecular dynamics simulation, indicated that DHT and PIK3CA bound narrowly and steadily. Moreover, the binding free energy of PIK3CA_DHT was −28.64 ± 0.89 kcal/mol. A negative value indicated that the two molecules had a binding affinity with the target protein, and an inferior value indicated a more solid binding. Our findings indicated the binding affinity of PIK3CA_DHT was considerably strong. PIK3CA encrypts the p110 catalytic subunit of PI3K, namely PI3K P110α (Zhang et al., 2020). Through the PI3K-AKT signaling network, PIK3CA plays a biological role in various pathologies, such as tumors (Mei et al., 2016), vascular malformation (Van Damme et al., 2020), diabetic cardiomyopathy (Prakoso et al., 2022). For breast cancer, there is currently the PIK3CA drug Alpelisib, which is an oral PI3K inhibitor. It can inhibit the activity of PIK3CA and has synergistic anti-tumor activity when combined with Fulvestrant (Rugo et al., 2021). However, there are still few studies on PIK3CA effects on DKD.

In our study, we found that DHT could have a beneficial effect on DKD via PIK3CA by regulating the PI3K-AKT signaling network, which plays a significant part in glucose metabolism, differentiation, cell growth, proliferation, and apoptosis, among other cellular roles (Zeng et al., 2019). This signaling pathway has an elevated expression in many renal injured tissues. (Xu et al., 2021). In particular, the incidence and progress of DKD are tightly associated with the over-activation of the PI3K-AKT signaling network It has already been reported that the inhibition of the PI3K-AKT signaling pathway can enhance podocyte autophagy and delay the occurrence of DKD (Yang et al., 2020). Moreover, TGF-β1 over-activates the PI3K-AKT signaling network and participates in transforming renal tubular epithelial cells into mesenchymal cells (EMT) (Kattla et al., 2008). In the case of triptolide, it alleviates high glucose-induced EMT in HK-2 cells by reducing the overactivation of the PI3K-AKT signaling network (Xue et al., 2018). The inhibition of the PI3K-AKT signaling network was reported to decrease the extracellular matrix accumulation in HMCs of STZ-induced rats (Zang et al., 2019). Lately, TCM berberine was shown to regulate HMCs proliferation and cell cycle through the PI3K-AKT signaling network to improve DKD (Ni et al., 2022). In light of all this, regulation of PI3K-AKT signaling network may be an encouraging medicinal approach for DKD.

In order to further clarify whether DHT improves the phenotypic switch of HMCs by regulating the PI3K-AKT signaling network through PIK3CA, we first examined the effect of DHT on HMCs viability with a concentration gradient. The results showed that DHT within 5 μM did not affect said viability, and the increase in DHT concentration improved the high glucose-induced phenotypic switching of HMCs. Our data illustrated that a-SMA was lower in the HG+DHT group compared with the HG group, and the over-trigger of PI3K-AKT signaling pathway was inhibited. After the administration of PIK3CA siRNA, the influence of DHT on the PI3K-AKT signaling system was eliminated. By molecular dynamics simulation, we ventured that DHT improves the phenotypic switching of HMCs by regulating the PI3K-AKT signaling system through binding to PIK3CA. Through *in vivo* research, we found that DHT can noticeably reduce the level of proteinuria and improve glomerular hypertrophy. Therefore, DHT may be a promising PIK3CA inhibitor for DKD and is worthy of further study.

Our research may provide new strategies for the molecular mechanisms of SM as a therapeutic agent for DKD. However, our work has some limitations. First, we screened the active components of SM through a subjective threshold, which entails the possibility of ignoring other relevant compounds. Second, the findings of the single chip analysis could result in a high false positive rate. Therefore, it is essential to increase the detection ability through the integration of multiple data sets. Third, in order to clarify whether DHT is beneficial to the outcome of DKD patients, evaluations on the safety of this drug are necessary.

## 5 Conclusion

The present findings could have relevant implications for upcoming studies. Initially, we identified the pharmacological pathway of SM that influences DKD treatment. Then, PIK3CA proved to be the main target of SM in DKD treatment. Finally, as an active component of SM, DHT could be combined with the active pocket of PIK3CA to become a new PIK3CA inhibitor. Small-molecule medications (for instance, Alpelisib) are of general use in health institutions. Therefore, we suggest that DHT is a promising candidate for the treatment of DKD. Our results support the application value of transcriptomics combined with network pharmacology in DKD research. Furthermore, our findings may offer insights towards drug innovation and additional drug academic investigations.

## Data Availability

The datasets presented in this study can be found in online repositories. The names of the repository/repositories and accession number(s) can be found in the article/Supplementary Material.

## Abbreviations

DKD, diabetic kidney disease; HMCs, human mesangial cells; SCr, serum creatinine; BUN, blood urea nitrogen; KEGG, Kyoto Encyclopedia of Genes and Genomes; GO, Gene Ontology; MDS, molecular dynamics simulation; TCM, Traditional Chinese Medicine; SM, Salvia miltiorrhiza; DHT, Dehydromiltirone; PIK3CA, phosphatidylinositol-4,5-bisphosphate 3-kinase catalytic subunit alpha; PPI, Protein-protein interaction; FBS, Fetal bovine serum; NS, normal saline; PI3K, phosphatidylinositol-3-hydroxykinase; AKT, protein kinase B; siRNA, small interfering RNA; CCK-8, Cell Counting Kit-8; ECM, extracellular matrix; Col-I, Collagen I; α-SMA, α-smooth muscle actin; FN, Fibronectin.
